# Understanding older patients’ self-management abilities: functional loss, self-management, and well-being

**DOI:** 10.1007/s11136-012-0131-9

**Published:** 2012-02-17

**Authors:** J. M. Cramm, J. M. Hartgerink, E. W. Steyerberg, T. J. Bakker, J. P. Mackenbach, A. P. Nieboer

**Affiliations:** 1 Institute of Health Policy and Management (iBMG), Erasmus University, Burgemeester Oudlaan 50, 3062 PA Rotterdam, The Netherlands; 2 Department of Public Health, Erasmus University Medical Centre, Rotterdam, The Netherlands; 3 ARGOS Zorggroep, Schiedam, The Netherlands

**Keywords:** Abilities, Quality of life, Self-management, Well-being, Older people, Functioning

## Abstract

**Purpose:**

This study aimed to increase our understanding of self-management abilities and identify better self-managers among older individuals.

**Methods:**

Our cross-sectional research was based on a pilot study of older people who had recently been admitted to a hospital. In the pilot study, all patients (>65 years of age) who were admitted to the Vlietland hospital between June and October 2010 were asked to participate, which led to the inclusion of 456 older patients at baseline. A total of 296 patients (65% response rate) were interviewed in their homes 3 months after admission. Measures included social, cognitive, and physical functioning, self-management abilities, and well-being. We used descriptive, correlations, and multiple regression analyses. In addition, we evaluated the mediation effect of self-management abilities on well-being.

**Results:**

Social, cognitive, and physical functioning significantly correlated with self-management abilities and well-being (all *p* ≤ 0.001). After controlling for background characteristics, multiple regression analysis indicated that social, cognitive, and physical functioning still related to self-management abilities (*β* = 0.17–0.25; all *p* ≤ 0.001). Older people with low levels of social, cognitive, and physical functioning were worse self-managers than were those with higher levels of functioning.

**Conclusions:**

Self-management abilities mediate the relationship between social, cognitive, and physical functioning and well-being. Interventions to improve self-management abilities may help older people better deal with function losses as they age further.

## Introduction

Hospitalized older patients are at risk of functional loss [1–4]. Among 70-year-olds, 35% showed some loss of function upon discharge compared with their pre-admission status; this rose to 65% for persons aged 90 years or older [2]. Although hospital-related functional loss among older people is often associated with complications of an illness or its treatment [1], it is only partially so [2–4], implying that the hospital stay per se is a contributor. Wu and colleagues [3] found that one or more limitations developed within 2 months in 42% of older patients with no baseline dependency at admission. Sager and colleagues [4] found that the ability to perform one or more activities of daily living had declined in 32% of older patients at the time of discharge. Functional loss may lead to readmission, prolonged hospital stay, transfer to a nursing home, or early death [5, 6]. Furthermore, it leads to poor well-being outcomes, greater dependence and thus higher burden on informal caregivers [7–9], higher utilization of healthcare, and, in turn, higher healthcare costs [10]. Preventing or reducing functional loss at an early stage of risk to maintain well-being of older people is therefore important [11].

**Fig. 1:**
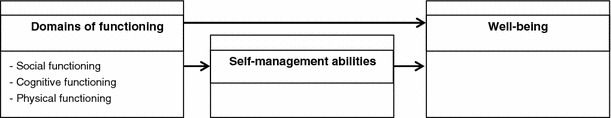
Theoretical model of functioning, self-management abilities, and well-being

Self-management abilities are expected to mediate the negative effect of declines in these domains of functioning on well-being [12–14]. Self-management abilities become thus particularly important in the face of loss of function. Health- or disease-related self-management abilities (taking medication, exercise, eating healthy, quit smoking) have been developed and translated effectively into interventions [15, 16]. In addition to health-related self-management abilities, there may also be a need for interventions aimed at the self-management of overall health and well-being to contribute to the (pro)active creation and maintenance of one’s own health and well-being. A substantial number of older patients suffer from a mixture of problems in multiple life domains; successful aging not only concerns physical health, but also involves social and psychological well-being [17–19]. Therefore, they may benefit more from self-management interventions that provide them with a general cognitive and behavioral repertoire for dealing with different kinds of problems rather than from interventions focusing on disease or health-related problems only. Relatively few interventions are designed to explicitly focus on the achievement and maintenance of well-being. The Self-Management of Well-being (SMW) theory [20], which is based on the theory of social production functions (SPF) [21, 22], offers concrete guidelines for the achievement of better self-regulation with regard to well-being. The SMW theory distinguishes six self-management abilities: (1) having a positive frame of mind, (2) being self-efficacious, (3) taking initiative, (4) investing in resources for long-term benefits, (5) taking care of a variety of resources, and (6) taking care of resource multifunctionality. Self-management abilities to achieve and maintain well-being depend on whether older people have adequate levels of social, cognitive, and physical functioning for fulfilling their well-being needs and goals [23]. As such, lower levels of functioning are expected to result in poorer self-management abilities. Poorer levels of social, cognitive, and physical functioning, for example, could negatively affect the self-management ability ‘having a positive frame of mind,’ which refers to the ability to adopt and maintain a positive frame of mind or positive expectations. The ability to have a positive frame of mind is expected to contribute to well-being because it extends the time horizon and boosts confidence, which, in turn, encourages people to engage in activities and not to give up easily [20]. Lower levels of social, cognitive, and physical functioning might lead to negative thoughts, feelings, and lower levels of confidence and motivation, which are expected to harm the self-management abilities ‘having a positive frame of mind’ and ‘taking initiative.’ In addition, they might negatively affect the ability to be self-efficacious and to gain and maintain a belief in personal competence, which is important to maintain well-being [23, 24]. Investment behavior is important for the realization and maintenance of well-being, even among older people with a declining time horizon [20]. Without investment behavior, there will be a (stronger) decline in social, cognitive, and physical functioning and well-being. Kahana and colleagues [25], for example, found that proactive prevention activities in older people have positive consequences for longevity and well-being. The self-management ability taking care of a variety of resources refers to having more than one resource or activity to achieve a specific aspect of well-being, for example, having a spouse, siblings, and friends as resources for affection. The primary importance of having a variety of resources lies in its buffer function to maintain well-being, since a variety of resources implies that there are possibilities to compensate loss [26]. Function declines in social, cognitive, and physical functioning may reduce buffer function to maintain well-being. Taking care of resource multifunctionality refers to activities that serve multiple aspects of well-being (e.g., social and physical well-being) simultaneously and in a mutually reinforcing way, for example, going for a walk (physical well-being) with friends (social well-being). Poorer levels of social, cognitive, and physical functioning may limit opportunities for multifunctionality, which is expected to negatively affect well-being. Many older people experience losses in social, cognitive, and physical functioning that may affect their self-management abilities; thus, self-management interventions may best be aimed at older people at risk of functional loss. This is supported by the findings of Schuurmans and colleagues [27] that frailty is strongly related to a decline in self-management abilities. Research investigating the relationship between levels of functioning and self-management among older people at risk of function loss is scarce. Understanding self-management abilities among those older people and identifying poor self-managers could be a path to mitigating age-related functional declines and subsequent deteriorations in well-being. Therefore, this research aimed to identify better self-managers among older individuals at risk of function loss by examining the relationship between social, cognitive, and physical functioning and self-management abilities, which in turn can mediate the relationship between social, cognitive, and physical functioning and well-being (Fig. 1). We thus aimed to (1) identify the role of social, cognitive, and physical functioning on self-management abilities and well-being among older people vulnerable to functional loss due to hospitalization and (2) determine the mediating role of self-management abilities in the relationship between social, cognitive, and physical functioning and well-being.

## Methods

### Study population

Our cross-sectional research was based on a pilot study of older people who had recently been admitted to a hospital. The results of the pilot study have been used to identify possible practical implementation problems in preparation for the main evaluation study and serve as a base for power calculations for the main study [28]. In the pilot study, all patients (>65 years of age) who were admitted to the Vlietland hospital between June 2010 and October 2010 were asked to participate, which led to the inclusion of 456 older patients at baseline (within 48 h after hospital admission). A total of 296 patients (65% response rate) were interviewed in their homes 3 months after admission. Exclusion reasons were as follows: lost interest to participate (*n* = 52), too ill (*n* = 35), terminally ill (*n* = 5), objection by partner/family (*n* = 14), mentally not able (*n* = 8), private reasons (e.g., death of spouse; *n* = 4), questions not applicable (*n* = 8), no contact/unable to reach respondent (*n* = 12), and reason unknown (*n* = 22). Deceased patients were excluded from the study sample (*n* = 49). The study protocol was approved by the medical ethics committee of the Erasmus Medical Centre, Rotterdam, the Netherlands, under protocol number MEC2011-041. Informed consent was obtained from all participants.

### Measures

- *Well*-*being* (the outcome variable) was measured with the 15-item version of the Social Production Function Instrument for the Level of Well-being [SPF-IL(s)] [29]. This instrument is based on the SPF theory and contains both physical and social well-being. For physical well-being, two basic needs are specified: comfort and stimulation. Social well-being is achieved through the fulfillment of three basic social needs: affection, behavioral confirmation, and status. Answers could be given on a four-point scale, ranging from never (1) to always (4). A higher score indicates greater well-being. An overall sumscore was used, with higher scores indicating higher levels of well-being.
- *Self*-*management* was measured with the 30-item Self-Management Abilities Scale (SMAS), which consists of six five-item subscales [24]. The subscales taking initiative, investing, self-efficacy, variety, and multifunctionality are related to the physical and social dimensions of well-being, while the ability to have a positive frame of mind is considered to be a more general cognitive frame [24]. Examples of self-management abilities are investing in resources for long-term benefits, efficaciously managing resources, and taking initiatives (i.e., being instrumental or self-motivating in enhancing health and well-being). Average self-management ability scores ranged from 1 to 5, with higher scores indicating higher self-management abilities.
- *Social functioning* was measured using the social component of the Short Form 20 Health Survey (SF-20). This social functioning scale focuses on whether the respondent’s health has limited social activities. The scale was transformed to range from 0 to 100, with higher scores indicating higher levels of social functioning.
- *Cognitive functioning* was assessed with the Mini Mental State Examination (MMSE), which measures cognitive functioning via interviews in which patients are asked questions about orientation in time and space, short- and middle-term memory, comprehension, and other cognitive dimensions. Scores ranged from 0 to 30, with higher scores indicating higher levels of cognitive functioning. Any score ≥25 points (of 30) represents effective cognitive functioning (intact). Below this, scores can indicate severe (≤9 points), moderate (10–20 points), or mild (21–24 points) cognitive functioning losses [30, 31].
- *Physical functioning* was assessed using the Katz Index of independence in activities of daily living [32, 33], which ranks an individual’s ability to perform six functions: bathe, dress, use the toilet, transfer, remain continent, and feed oneself. Scores of yes (1) or no (2) indicate (in)dependence in each function, with 6 = full physical function, 4 = moderate, and ≤2 = severe physical function impairment.
- *Education* ranged from 1 (no school or some primary education; <6 years) to 7 (university degree; >18 years).
- *Age*, *gender,* and *marital status* were also assessed.

### Analysis

Descriptive analysis included calculating means and standard deviations (SDs). The mediation effect of self-management abilities on well-being was evaluated based on conditions put forth by Baron and Kenny [34, 35] and Judd and Kenny [36].

- Condition 1: The theoretically specified *independent variables* (social, cognitive, and physical functioning) must emerge as significant predictors of the *outcome variable* (well-being) in correlation analyses.
- Condition 2: The theoretically specified independent variables must emerge as significant predictors of the *mediator variable* (self-management abilities) in correlation analyses.
- Condition 3: The mediator variable must be significantly associated with the outcome variable after controlling for the independent variables.
- Condition 4: The relationship between the *significant* independent variables and the outcome variable (well-being) must be significantly reduced when the effects of the mediator variable (self-management abilities) are included in the model.

After calculating bivariate correlations to investigate conditions 1 and 2, multiple regression analyses were performed to assess conditions 3 and 4. In addition, Steiger’s *Z* tests were used to test whether coefficients were significantly reduced when the effects of the mediator variable (self-management abilities) were included in the model [37]. All statistical analyses were conducted with SPSS software (ver. 17.0; SPSS, Inc., Chicago, IL, USA).

## Results

Respondents had a median age of 75.8 years (SD = 6.8 years; range = 65–94 years; Table 1). About half (54.2%) were women, just over half (56.6%) were married/partnered, and 43.4% were single, widowed, or divorced. Most (55.9%) lived independently with others; about one-third (37.3%) lived independently alone, and 6.8% lived in senior residences or nursing homes. The mean educational level was 4.1 (SD = 1.6; range = 1–7). The mean well-being score of our sample (2.8; SD = 0.4; range = 1.3–3.8) was comparable to that measured by Frieswijk and colleagues [38] using the SPF-IL among slightly to moderately frail older people (mean = 2.8; SD = 0.4).

**Table 1:** Characteristics of the study population

	[Mean ± SD (range)] or %	*n*
*Background characteristics*		
Age	75.8 ± 6.8 (65–94)	291
Gender (female)	45.8%	295
Marital status (married/living together)	56.6%	295
Education	4.1 ± 1.6 (1–7)	295
*Functioning domains*		
Social functioning (SF-20)	68.6 ± 31.7 (0–100)	293
Cognitive functioning (MMSE)	28.1 ± 2.0 (19–30)	293
Physical functioning (Katz)	5.6 ± 0.8 (1–6)	293
*Self*-*management abilities* (SMAS)	2.7 ± 0.8 (0.2–4.8)	282
*Well*-*being* (SPF-IL)	2.8 ± 0.4 (1.3–3.8)	288

*SD* standard deviation, *SF-20* Short Form 20 Health Survey, *MMSE* Mini Mental State Examination, *Katz* Katz index of independence in activities of daily living, *SMAS* Self-Management Abilities Scale, *SPF-IL* Social Production Function Instrument for the Level of Well-being

Correlations of independent variables with well-being are displayed in Table 2. The results indicated that functioning (social, cognitive, and physical) was significantly related to well-being (all *p* ≤ 0.001). Self-management abilities were strongly associated with social, cognitive, and physical functioning, as well as with well-being (all *p* ≤ 0.001). Thus, our results met conditions 1 and 2.

**Table 2:** Correlations among background characteristics, domains of functioning, self-management abilities, and well-being in older people at risk of function loss (*n* = 296)

	1.	2.	3.	4.	5.	6.	7.	8.
1. Age								
2. Gender (female)	0.13*							
3. Marital status (married)	−0.20***	−0.34***						
4. Education (1–7)	0.03	0.09	0.03					
5. Social functioning (SF-20)	−0.12*	−0.07	0.06	−0.04				
6. Cognitive functioning (MMSE)	−0.20***	0.02	0.15**	0.12*	0.09			
7. Physical functioning (Katz)	−0.25	−0.22***	0.22***	−0.02	0.34***	0.14**		
8. Self-management abilities (SMAS)	−0.13*	0.15**	0.07	0.05	0.32***	0.23***	0.31***	
9. Well-being (SPF-IL)	−0.01	0.03	0.06	0.02	0.44***	0.22***	0.31***	0.65***

*SF-20* Short Form 20 Health Survey, *MMSE* Mini Mental State Examination, *Katz* Katz index of independence in activities of daily living, *SMAS* Self-Management Abilities Scale, *SPF-IL* Social Production Function Instrument for the Level of Well-being

*** *p* ≤ 0.001; ** *p* ≤ 0.01; * *p* ≤ 0.05 (two-tailed)

Table 3 displays the results of the multiple regression analyses using the mediating variable (self-management) as the dependent variable. After controlling for background characteristics, the results indicated that social (*β* = 0.22; *p* ≤ 0.001), cognitive (*β* = 0.17; *p* ≤ 0.001), and physical (*β* = 0.25; *p* ≤ 0.001) functioning were all significantly related to self-management abilities.

**Table 3:** Relationships among background characteristics, domains of functioning, and self-management abilities of older people, as determined by multiple regression analyses (standardized *β*)

Self-management abilities scale (SMAS)	
*Background characteristics*	
Age	−0.02
Gender (female)	0.24***
Marital status (married/living together)	0.04
Education (1–7)	0.01
*Domains of functioning*	
Social functioning (SF-20)	0.22***
Cognitive functioning (MMSE)	0.17**
Physical functioning (Katz)	0.25***
Adjusted R² for equation	21.0%
F-value	11.512

*SF-20* Short Form 20 Health Survey, *MMSE* Mini Mental State Examination, *Katz* Katz index of independence in activities of daily living

*** *p* ≤ 0.001; ** *p* ≤ 0.01; * *p* ≤ 0.05 (two-tailed)

Multiple regression analyses were performed to test conditions 3 and 4. Table 4 shows the direct effects of background characteristics and social, cognitive, and physical functioning on the outcome variable (well-being). After controlling for all independent variables, self-management abilities significantly affected well-being (*β* = 0.56; *p* ≤ 0.001), thus meeting condition 3.

**Table 4:** Multiple regression analysis of background characteristics, domains of functioning, and the mediating effect of self-management abilities on the well-being (SPF-IL) of older people at risk of function loss

	Adjusted *R* ^²^	*F*-value	*β* (step 1)	*β* (step 2)
*Background characteristics (step 1)*	23%	12.89***		
Age			0.11	0.12
Gender (female)			0.10	−0.04
Marital status (married)			0.02	0.00
Education (1–7)			−0.03	−0.03
*Domains of functioning*				
Social functioning (SF-20)			0.34***	0.22***
Cognitive functioning (MMSE)			0.17***	0.08
Physical functioning (Katz)			0.22***	0.09
*Mediator (step 2)*	48%	32.77***		
*Self*-*management abilities* (SMAS)				0.562***

*SF-20* Short Form 20 Health Survey, *MMSE* Mini Mental State Examination, *Katz* Katz index of independence in activities of daily living, *SMAS* Self-Management Abilities Scale, *SPF-IL* Social Production Function Instrument for the Level of Well-being

*** *p* ≤ 0.001; ** *p* ≤ 0.01; * *p* ≤ 0.05 (two-tailed)

In step 1 of the regression model, social (*β* = 0.34; *p* ≤ 0.001), cognitive (*β* = 0.17; *p* ≤ 0.001), and physical (*β* = 0.22; *p* ≤ 0.001) functioning significantly affected the well-being of older people at risk of function loss. To meet condition 4, the relationship between social, cognitive, and physical functioning and the outcome variable (well-being) must be significantly reduced when the effects of the mediator (self-management abilities) are included in the model. Thus, self-management abilities were included in step 2 of the regression analysis. Social functioning remained significantly related to well-being (*β* = 0.22; *p* ≤ 0.001), but the strength of the relationship diminished from *β* = 0.34 to *β* = 0.22 (*z* = 2.15; *p* ≤ 0.01). The same pattern was observed for the other domains. The strengths of the relationships of well-being with cognitive (*β* = 0.17 versus *β* = 0.08) declined significantly (*z* = 1.68; *p* ≤ 0.05) and that with physical (*β* = 0.22 vs. *β* = 0.09) functioning also declined significantly (*z* = 2.24; *p* ≤ 0.01). Cognitive and physical functioning were not significantly associated with well-being when self-management abilities were included in the equation. Thus, self-management abilities acted as mediators between social, cognitive, and physical functioning and well-being among older people recently admitted to hospital and at risk of function loss. Furthermore, cognitive and physical functioning are completely mediated, whereas social functioning is only partially mediated by self-management abilities.

## Discussion

This study aimed to identify the role of social, cognitive, and physical functioning on self-management abilities and well-being among older people vulnerable to functional loss due to hospitalization. We also examined whether self-management abilities mediate the relationship between social, cognitive, and physical functioning and well-being. Our results showed that levels of social, cognitive, and physical functioning were indeed strongly related to self-management abilities. This implies that older people with low levels of social, cognitive, and physical functioning are worse self-managers than are those with higher levels of functioning. In addition, social, cognitive, and physical functioning were also strongly related to well-being. Such results align with those of previous studies, which have found that multiple domains of functioning affect well-being [38, 39]. Furthermore, this study showed the mediating role of self-management abilities in the relationship between social, cognitive, and physical functioning and well-being. Enhancing self-management abilities of at-risk older people is thus critical. Special attention may be needed for older patients reporting low levels of social, cognitive, or physical functioning who are worse self-managers than more highly functioning older people. These patients may benefit from case-management attention to enhance self-management abilities to prevent further—and hospital-induced—loss of function. We also found that whereas cognitive and physical functioning were completely mediated, social functioning was only partially mediated by self-management abilities. In part, this may be the result of the strong relationship between social functioning and well-being. A meta-analysis provided evidence to support the directional influence of social relationships on mortality [40], which may also hold for well-being. Correlational analyses indeed showed a stronger relationship between social functioning and well-being compared to the relationship between well-being, physical, and cognitive functioning. Furthermore, while physical and cognitive functioning primarily depend on the person, social functioning may also depend on the abilities of other people in one’s social network. The abilities of others may play an important role in maintaining one’s social relationships. This may explain why social functioning was only partially mediated by self-management abilities of the older persons.

Our findings are based on a pilot study conducted in 2010 among older people who had recently been admitted to a hospital in the context of the Prevention and Reactivation Care Programme [28]. The program supports a multifaceted and multidisciplinary case-management approach to the care of older individuals organized around several core components, including screening for vulnerability and early detection and treatment of (functional) health problems. The main goal of the program is to reduce the loss of function among older patients and the burden on the caregiver during and after hospital discharge. Investigation of and attention to the self-management abilities of recently hospitalized older people are thus necessary.

Examples of self-management interventions for older people are education on lifestyle, regulatory skills, and proactive coping. In addition, interventions on mood disorders (depression, anxiety, and aggression) in combination with caregiver support [41] are other important promising developments. However, older patients’ abilities to self-manage their social lives and activities, such as regularly socializing with family and friends and being physically active, must also be addressed. Interventions that aim to enhance self-management abilities may provide a useful addition to traditional interventions, which focus solely on the physical decline associated with aging and chronic conditions [18, 19, 38, 42, 43]. Kremers and colleagues [42] showed that a self-management group intervention based on the SMW theory improved self-management ability and well-being in single older women. Two other empirical studies [38, 43] have shown improvement in overall self-management ability (vs. control groups) through the implementation of bibliotherapy and home-based training interventions. These improvements remained significant after 6 months for bibliotherapy [38] and 4 months for individual home-based training [43]. Both interventions also showed significant improvements in four of the six self-management abilities (self-efficacy, taking initiative, resource investment, and resource variety), but not in positive frame of mind or resource multifunctionality. These studies, however, were conducted among frail older people in the community. Older people at risk of function loss due to hospitalization may also benefit from interventions that enhance self-management abilities.

The limitations of this study should be considered when interpreting the findings. Most importantly, the data collected were cross-sectional, and therefore, causal relationships could not be inferred. While our study showed that self-management abilities are important to mediate the relationship between social, cognitive, and physical functioning and well-being of older people at risk of function loss, we did not investigate whether interventions aiming to enhance these abilities actually did improve self-management. Further research is necessary to explore ways in which the self-management abilities of older people at risk of function loss due to hospitalization can be improved. Finally, our study sample consisted of older people who had recently been admitted to a hospital, which limits generalizability of our study findings.

## Conclusions

We conclude that older people with low levels of social, cognitive, and physical functioning are worse self-managers than are those with higher levels of functioning. We also identified the mediating role of self-management abilities in the relationship between social, cognitive, and physical functioning and well-being. Interventions to improve self-management abilities may help older people better deal with function losses as they age further. We feel these results provide a useful basis for the design of effective interventions for successful aging among older people at risk of functional loss.

## References

[1] Creditor MC (1993). Hazards of hospitalization of the elderly. Annals of Internal Medicine.

[2] Covinsky KE, Palmer RM, Fortinsky RH, Counsell SR, Stewart AL, Kresevic D (2003). Loss of independence in activities of daily living in older adults hospitalized with medical illnesses: Increased vulnerability with age. Journal of the American Geriatrics Society.

[3] Wu AW, Yutaka Y, Alzola C (2000). Predicting functional status outcomes in hospitalized patients aged 80 years and older. Journal of the American Geriatrics Society.

[4] Sagar MA, Franke T, Inouye SK (1996). Functional outcomes of acute medical illness and hospitalization in older persons. Archives of Internal Medicine.

[5] Boyd CM, Landefeld CS, Counsell SR, Palmer RM, Fortinsky RH, Kresevic D (2008). Recovery of activities of daily living in older adults after hospitalization for acute medical illness. Journal of the American Geriatrics Society.

[6] de Rooij SE, Govers A, Korevaar JC, Abu-Hanna A, Levi M, de Jonge E (2006). Short-term and long-term mortality in very elderly patients admitted to an intensive care unit. Intensive Care Medicine.

[7] Covinsky KE, Justice AC, Rosenthal GE, Palmer RM, Landefeld CS (1997). Measuring prognosis and case mix in hospitalized elders. The importance of functional status. Journal of General Internal Medicine.

[8] Covinsky KE, Wu AW, Landefeld CS, Connors AF, Phillips RS, Tsevat J (1999). Health status versus quality of life in older patients: Does the distinction matter?. American Journal of Medicine.

[9] Inouye SK, Peduzzi PN, Robison JT, Hughes JS, Horwitz RI, Concato J (1998). Importance of functional measures in predicting mortality among older hospitalized patients. Journal of the American Medical Association.

[10] McCusker J, Kakuma R, Abrahamowicz M (2002). Predictors of functional decline in hospitalized elderly patients: A systematic review. The Journals of Gerontology, Series A: Biological Sciences and Medical Sciences.

[11] Health Council of the Netherlands. (2009). *Prevention in the elderly: Focus on functioning in daily life*. Publication no. 2009/07. The Hague: Health Council of the Netherlands.

[12] Bandura A (1997). Self-efficacy: The exercise of control.

[13] Marino P, Sirey JA, Raue PJ, Alexopoulos GS (2008). Impact of social support and self-efficacy on functioning in depressed older adults with chronic obstructive pulmonary disease. International Journal of Chronic Obstructive Pulmonary Disease.

[14] Wu, L. M., Austin, J., Hamilton, J. G., Valdimarsdottirm, H., Isolam, L., Rowley, S., et al. (2011). Self‐efficacy beliefs mediate the relationship between subjective cognitive functioning and physical and mental well‐being after hematopoietic stem cell transplant. *Psycho*-*Oncology*. doi:10.1002/pon.2012. 10.1002/pon.2012 PMC3788830 21739524

[15] Clark NM, Janz NK, Becker MH, Schork MA, Wheeler J, Liang J, Dodge JA, Keteyian S, Rhoads KL, Santinga JT (1992). Impact of self-management education on the functional health status of older adults with heart disease. Gerontologist.

[16] Lorig K, Sobel DS, Stewart AL, Brown BW, Bandura A, Ritter P, Gonzalez VM, Laurent DD, Holman HR (1999). Evidence suggesting that a chronic disease self-management program can improve health status while reducing hospitalization. A randomized trial. Medical Care.

[17] Baltes PB, Baltes MM, Baltes PB, Baltes MM (1990). Psychological perspectives on successful aging: The model of selective optimization with compensation. Successful aging: perspectives from the behavioral sciences.

[18] Rowe JW, Kahn RL (1987). Human aging: Usual and successful. Science.

[19] Rowe JW, Kahn RL (1997). Successful aging. Gerontologist.

[20] Steverink N, Lindenberg S, Slaets JPJ (2005). How to understand and improve older people’s self-management of wellbeing. European Journal of Ageing.

[21] Lindenberg, S. (1996). Continuities in the theory of social production functions. In H. Ganzeboom, & S. Lindenberg (Eds.), *Verklarende sociologie* [Explanatory sociology] (pp. 169–184). Amsterdam: Thesis Publishers.

[22] Steverink N, Lindenberg S, Ormel J (1998). Towards understanding successful ageing: Patterned change in resources and goals. Ageing & Society.

[23] Steverink N, Lindenberg S (2008). Do good self-managers have less physical and social resource deficits and more well-being in later life?. European Journal of Ageing.

[24] Schuurmans H, Steverink N, Frieswijk N, Buunk BP, Slaets JPJ, Lindenberg S (2005). How to measure self-management abilities in older people by self-report? The development of the SMAS-30. Quality of Life Research.

[25] Kahana E, Lawrence RH, Kahana B, Kercher K, Wisniewski A, Stoller E, Tobin J, Stange K (2002). Long-term impact of preventive proactivity on quality of life of the old–old. Psychosomatic Medicine.

[26] Nieboer A, Lindenberg S, Gullone E, Cummins RA (2002). Substitution, buffers and subjective well-being: A hierarchical approach. The universality of subjective well-being indicators.

[27] Schuurmans H, Steverink N, Lindenberg S, Frieswijk N, Slaets JP (2004). Old or frail: what tells us more?. Journal of Gerontology: medical sciences.

[28] Asmus-Szepesi KJ, de Vreede PL, Nieboer AP, van Wijngaarden JD, Bakker TJ, Steyerberg EW (2011). Evaluation design of a reactivation care program to prevent functional loss in hospitalised elderly: A cohort study including a randomised controlled trial. BMC Geriatrics.

[29] Nieboer A, Lindenberg S, Boomsma A, van Bruggen AC (2005). Dimensions of well-being and their measurement: The SPF-IL scale. Social Indicators Research.

[30] Folstein MF, Folstein SE, McHugh PR (1975). “Mini-mental state”. A practical method for grading the cognitive state of patients for the clinician. Journal of Psychiatric Research.

[31] Kempen GI, Brilman EI, Ormel J (1995). The Mini Mental Status Examination. Normative data and a comparison of a 12-item and 20-item version in a sample survey of community-based elderly. Tijdschrift voor Gerontologie en Geriatrie.

[32] Katz S, Ford AB, Moskowitz RW, Jackson BA, Jaffe MW (1963). The Index of ADL: A standardized measure of biological and psychosocial function. Journal of the American Medical Association.

[33] Katz S, Down TD, Cash HR, Grotz RC (1970). Progress in the development of the index of ADL. Gerontologist.

[34] Baron RM, Kenny DA (1986). The moderator-mediator variable distinction in social psychological research: Conceptual, strategic, and statistical considerations. Journal of Personality and Social Psychology.

[35] Kenny, D. A. (2010). Mediation. Baron and Kenny Steps. Accessed March 2010. http://davidakenny.net/cm/mediate.htm#BK.

[36] Judd CM, Kenny DA (1981). Process analysis: Estimating mediation in treatment evaluations. Evaluation Review.

[37] Meng XL, Rosenthal R (1992). Comparing correlated correlation coefficients. Psychological Bulletin.

[38] Frieswijk N, Steverink N, Buunk BP, Slaets JPJ (2006). The effectiveness of a bibliotherapy in increasing the self-management ability of slightly to moderately frail older people. Patient Education and Counseling.

[39] Nieboer AP, Schulz R, Matthews KA, Scheier MF, Ormel J, Lindenberg SM (1998). Spousal caregivers’ activity restriction and depression: A model for changes over time. Social Science and Medicine.

[40] Holt-Lunstad J, Smith TB, Layton JB (2010). Social relationships and mortality risk: A meta-analytic review. PLoS Medicine.

[41] Bakker TJ, Duivenvoorden HJ, van der Lee J, Olde Rikkert MG, Beekman AT, Ribbe MW (2010). Integrative psychotherapeutic nursing home program to reduce multiple psychiatric symptoms of cognitively impaired patients and caregiver burden: Randomized controlled trial. American Journal of Geriatric Psychiatry.

[42] Kremers IP, Steverink N, Albersnagel FA, Slaets JPJ (2006). Improved self-management ability and well-being in older women after a short group intervention. Aging and Mental Health.

[43] Schuurmans, H. (2004). Promoting well-being in frail elderly people: Theory and intervention. GRoningen Intervention Program (GRIP). Dissertation, University of Groningen. Available at: http://opc.ub.rug.nl. 15704605

